# Feeling Identified vs. Behaving as Such: A Multi-Study Project on Chinese Organizational Identification and Chinese Employees’ Identification Profiles

**DOI:** 10.3389/fpsyg.2019.01039

**Published:** 2019-06-11

**Authors:** Jie Yang, Hannah-Hanh D. Nguyen, Xiaobin Xiong, Xinyan Wang

**Affiliations:** ^1^Research Center for Innovation and Strategic Human Resource Management, Jiangxi University of Finance and Economics, Nanchang, China; ^2^Shidler College of Business, University of Hawai’i at Mānoa, Honolulu, HI, United States

**Keywords:** Chinese organizational identification, cultural studies, psychometrics scale development and validation, latent profile analyses, emic-etic research

## Abstract

We conducted a multi-study, field research program to (a) develop, validate and cross-validate an emic-etic, bi-dimensional measure of Chinese workers’ organizational identification (OID) based on our previously conceptualized framework, and (b) classify employees into three levels of OID. We found convergent evidence showing that the Chinese OID construct consists of emotional and behavioral dimensions. Specifically, in Study 1 (*N* = 408), we developed and validated a bi-dimensional measure called the Chinese Organizational Identification Questionnaire (COIQ; 8 items). In Study 2 (*N* = 299), we cross-validated the COIQ and established the construct validity by examining several hypothesized relationships between the Chinese OID construct and other relevant organizational variables, such as unethical pro-organizational behavior, perceived psychological contract violation, and perceptions of business practices of compensations and benefits. Based on the factor analytic and structural equation modeling results, we concluded that the bi-dimensional Chinese OID model as measured with the COIQ has construct validity. More importantly, we used the latent profile analysis method to generate three OID profiles of Chinese workers based on their COIQ scores: The Strong Identifier, the Moderate Identifier and the Action-Oriented Identifier. Those profiles were differentially related to the organizational constructs of interest. The implications for researchers and practitioners were discussed.

## Introduction

There is a crisis of employees’ commitment in Chinese firms: turnover rates are high in Chinese companies (Howard et al., 2007), implying that employees might not strongly identify with their organization. However, many Chinese workers still view the workplace as their family (Chen, 2001). Therefore, the Chinese organizational context is a fascinating opportunity to study Chinese workers’ organizational identification (OID) because some Chinese employees have experienced an organizational identity crisis and Chinese employers increasingly need to identify and retain their talents since this country has transformed from a central-planning economy to a socialist market economy (Nee, 1992).

According to Tsui (2006), it is essential to contextualize the study of Chinese management using an emic perspective: researchers should study a China’s context-specific management phenomenon important to Chinese companies and their employees, such as the OID concept in the present study. Therefore, we explored two research questions: To what extent do Chinese workers uniquely interpret the concept of OID? How can Chinese employers figure out which employee is most highly identified with their firm? To answer those questions, researchers first need a valid measurement tool of Chinese OID. In the past, some Chinese researchers (e.g., Li et al., 2008; Li and Yang, 2012) typically translated and/or adapted a well-known Western measurement instrument, such as Cheney’s (1983) 25-item Organizational Identification Questionnaire (OIQ), to assess Chinese workers’ OID. However, one concern about this practice is that Western measures might not fully capture the Chinese OID construct. Therefore, in the present study, we adopted Cheung et al.’s (2001) recommendation and used an emic-etic approach to develop an OID measure based partly on Chinese interpretation of the construct and partly on established measures in the literature as well: We first identified the indicators of the construct that might be applicable to only Chinese employees in addition to universal indicators similar to those found in the Western literature, and then validated the measure in a human resources management context.

## General Literature Review

### Organizational Identification Crisis in Chinese Organizations

According to Tsui (2004, 2006), non-Western organizational behavior researchers have conducted more indigenous research (i.e., collecting data from diverse cultural and ethnic groups) to interpret findings of an organizational phenomenon more accurately in a non-Western culture. The Chinese organizational context is unique due to the country’s complex socio-economic background. Specifically, Chinese organizations and institutions have faced a crisis of OID, including (a) a shortage of working capital, (b) the lack of organizational loyalty among skilled employees, and (c) the need for relying on personal and relational networks (Boisot and Child, 1999). For example, 38% of Chinese HR professionals reported a sharp increase of turnovers in their organization (Howard et al., 2007). The turnover rate among Chinese managers was 25% higher than the global average, and 30 to 40% of senior managers at multinational companies switched jobs every year (Gross and Heinold, 2005). It is because in the current labor market, loyalty and commitment-based employment in China has been replaced by a relationship of a mere economic exchange (Tsui and Wu, 2005).

### Organizational Identification Definition

#### Western Definitions

The concept of Western OID is rooted in the Social Identity Theory (Tajfel and Turner, 1986). According to Dutton et al. (1994), OID is the extent to which employees define themselves and their organization by the same attributes. A majority of OID researchers have defined the construct as an explicit cognitive process (see Rousseau, 1998). OID is also defined as the psychological connection or attachment a worker has with his or her organization (Edwards and Peccei, 2007; Ashforth et al., 2008; Xenikou, 2014).

#### A Chinese OID Conceptual Model

In the present paper, we defined OID among Chinese workers as a bi-dimensional construct consisting of mainly emotional and behavioral processes. This definition is generated from the results of a previous qualitative study (Yang and Nguyen, 2014) that we conducted to find an indigenous definition of Chinese OID based on Tsui’s “indigenization” approach (p. 8). Specifically, in that study, we adopted Glaser and Strauss (1967) grounded-theory qualitative method and systematically generated a theoretical model of Chinese OID based on the relationship of different themes emerging from the narrative text (see Bernard and Ryan, 1998). We first interviewed Chinese graduate students with work experience (*N* = 32) about their understanding of the nature of Chinese OID. Secondly, we analyzed the qualitative data and found that four OID themes emerged: (a) OID should be visible and observable (through one’s behavior); (b) OID has an emotional/affective component; (c) OID is an on-going process through which one aligns oneself with the organization, and (d) OID is about the similarity and attractiveness of an organization. We also asked participants what they thought would contribute to a higher level of OID; ten themes emerged from the responses and were subsequently categorized into two second-order categories based on the themes’ content: (a) “Organizational Upholding and Striving for Solidarity and Cooperation,” which refers to one’s concrete support for his or her organization by working toward a thought-action congruence with the organization values through their interactions and cooperation with their firm, and (b) “Emotional Attachment,” referring to the high identifiers’ feelings of belonging to their organization.

On the one hand, the conceptual framework of our OID definition partly reflects the universality of the construct: the Chinese theme of “organizational upholding” was consistent with Farh et al.’s (1997) operationalization of OID, which is “the employee’s effort to maintain the organizational image, active participation in organizational meetings and activities, and voluntary preposition of constructive solutions for organizational improvement” (p. 3770). One possible reason for the fact that Chinese participants emphasized the importance of “striving for solidarity and cooperation” among higher organizational identifiers (i.e., actively ensuring that the organization becomes more cohesive) is the influence of a “guanxi-oriented” culture (i.e., fundamentally relation-oriented) in Chinese organizational context and Chinese business community (Kao, 1993). For a worker who grows up with guanxi values, to identify with one’s organization is not to pay lip service but to act in support of the organization, such as keeping one’s solidarity with the organization, being loyal to one’s supervisors and colleagues, as well as being compliant in the workplace (see Redding, 1982; Lee, 1996).

On the other hand, our OID definition also points to the culture-specific aspect of the construct. Our qualitative findings showed that Chinese workers interpreted OID partly as an affective construct, whereas there was no distinct cognitive component typically common in the aforementioned Western OID theories. One possible explanation for this interesting finding is that Chinese respondents’ cultural values are closely associated with how they experience and express their emotions (see Bond, 1993). In other words, thoughts may be less important to Chinese workers than their feeling about their firm (and, subsequently, how they would act upon those feelings).

Another possible explanation comes from the literature of cross-cultural emotional management (e.g., Howard, 1993; Ashcraft, 2000). Whereas the rationality-emotionality *duality* is typically emphasized in Western companies (i.e., you should act rationally in the workplace, and being “emotional” is typically frowned upon as unprofessional), the rationality-emotionality *mutuality* is dominant in non-Western organizations (i.e., you should think with your head but act with your heart). In other words, Western organizations tend to bipolarize rationality and emotionality (i.e., employee thoughts and feelings being mutually exclusive); Western managers even promote thought over feeling and emotion. In contrast, non-Western companies accept that thoughts and feelings are intertwined and cannot be separated (Fineman, 2000). For example, Krone and Morgan (2000) surveyed Chinese managers and found that their thinking and feeling went hand in hand in a Chinese organizational context. Chinese managers would stress the importance of maintaining the continuity between thought and feeling because in the Chinese culture, the display of sensitivity to others is considered necessary for one’s spiritual development. Specifically, Krone and Morgan pointed out that, when handling negative events at work, it was essential for Chinese managers to “restore inner balance” (p. 92) and “use a heart to change a heart” (p. 93), meaning that managers need to reflect and understand their own emotional experience, as well as using positive emotional experiences to motivate their subordinates to work harder. In other words, according to Peng and Nisbett (1999), emotion is part of the homeostatic experience of Chinese workers (i.e., the mind, the body and the emotion working together in doing business); this type of experience reflects an indigenous Chinese holistic viewpoint.

In summary, the fact that we found a bi-dimensional conceptual framework of Chinese OID was consistent with some researchers’ position that OID is a multi-dimensional construct (e.g., Cheney, 1983; van Dick, 2001). Further, what makes our Chinese OID model unique is *the lack of a distinct cognitive component* typically characterizing a Western OID framework, and the presence of a rationality-emotionality mutuality core. Next, conducting a series of two follow-up quantitative studies, we aimed at validating the Chinese bi-dimensional model of OID with further empirical evidence in the present project.

### A Triangulation Research Program

Specifically, we expected that the construct of Chinese OID might share certain universal elements with those in the Western literature as well as having some unique Chinese aspects; therefore, we conducted an emic-etic research program to investigate the phenomenon. Our goal was to triangulate qualitative and quantitative evidence of the construct so that we could make “inferences from the sampling particulars of a study to the higher-order constructs they represent” (Shadish et al., 2002, p. 65). From the theory-building standpoint, our research program followed the *convergent methodology* (Campbell and Fiske, 1959) or *triangulation* (Denzin, 2012) commonly used in global management research and organizational research (e.g., Jick, 1979; Kiessling and Harvey, 2005; Swanson and Holton, 2005). In other words, we actively tested the Chinese OID construct not only by examining its structure but also by testing its relationships with other relevant organizational concepts.

#### Organizational Correlates

In the Western literature, OID was found to be positively related to positive attitudinal and affective outcomes in the workplace, such as affective organizational commitment (Riketta, 2005), job and organizational satisfaction (Knight and Haslam, 2010), job involvement (van Knippenberg and van Schie, 2000), organizational loyalty (Adler and Adler, 1988), occupational and work group attachment (Riketta and van Dick, 2005), and organizational change success and subjective well-being (Haslam et al., 2009). Moreover, a lower level of OID predicted more unethical organizational behaviors (Umphress et al., 2010) and greater turnover intention (Scott and Stephens, 2009). Recently, Callea et al. (2016) found that OID completely mediated the effect of job insecurity on job performance and organizational citizenship behavior.

Riketta (2005) conducted a meta-analysis of OID effects on various organizational outcomes and found that how OID was conceptualized and measured directly influenced its link to specific organizational outcomes. The implication is that the construct validity of a measurement instrument of OID should be evaluated by examining the qualities of the measurement tool, and the construct validity subsumes other validity evidences (e.g., content validity, criterion-related validity; see Messick, 1989). Therefore, we followed Messick’s best practices and looked for patterns of relationships among the COIQ and other organizational measures across samples.

#### Our Studies

In Study 1, we developed an emic-etic measure of Chinese OID with two dimensions (consistent with our aforementioned qualitative results from Yang and Nguyen, 2014), and we subsequently used factor analytic procedures to obtain the preliminary validity findings of the measure. In Study 2, we cross-validated the factor structure of this measurement instrument and then tested the construct validity of the Chinese OID framework by examining its conceptual relationships with other relevant organizational variables. Additionally, we used the OID data to establish general profiles of Chinese workers who variably identified themselves with their firm.

## Study 1 – Developing and Validating a Bi-Dimensional Measure of Chinese OID

### Introduction

#### Western OID Measures

Edwards (2005) reviewed the strengths and/or limitations of major OID instruments, such as those developed by Cheney (1983), Mael and Ashforth (1992), Miller et al. (2000), and Edwards and Peccei (2007). The author found that Cheney’s (1983) Organizational Identification Questionnaire (OIQ, 25 items) is the most commonly used measure in the literature. This measure assesses three components of OID: membership, loyalty and similarity. The measure yields acceptable to satisfactory internal consistency levels in the literature (e.g., Bullis and Bach, 1991; Johnson et al., 1996; Barker and Tompkins, 2010). Later, Miller et al. (2000) cross-validated the dimensions of Cheney’s (1983) OIQ, using multiple samples from different organizations and across time points. The researchers found that 12 items in Cheney’s OIQ represented a three-factor structure.

#### The Need for a Chinese OID Measure

To the best of our knowledge, Chinese OID researchers have mostly relied on a translated version of popular Western measurement tools (e.g., Li et al., 2008; Li and Yang, 2012). We deemed that this practice is not desirable because (a) there may be cultural differences and/or organizational context differences that might prevent Chinese respondents to fully interpret a Western measure of OID, and (b) we had previously found some empirical evidence that such Western measures might not capture the Chinese interpretation of OID. Specifically, in an unpublished pilot study, we tested those assumptions by cross-validating Miller et al.’s (2000) OIQ with two samples of Chinese employees (*N* = 403 in total). We ran a series of exploratory and confirmatory factor analyses (CFA) as well as bivariate correlation analyses and found that only a one-factor model (with 9 items) emerged and fit the Chinese data the best, which was inconsistent with the three-factor result in the Western literature. A possible reason is that Chinese culture is generally less future oriented, less assertive, more collectivistic (see Hofstede, 1980), and more rule-oriented than American culture as evidenced in a meta-analysis conducted by Javidan et al. (2006).

#### Measuring Chinese OID

Whereas we had followed Tsui’s (2006) call for indigenizing [OID] research in our previous qualitative theory-building study (Yang and Nguyen, 2014), in the present study, we followed Cheung et al.’s (2001) recommendation and used an emic-etic approach to develop an OID item pool to yield further inductive evidence for a Chinese OID framework for triangulation purpose. In other words, if a bi-dimensional conceptual framework of Chinese OID also emerges from the inductive research process in the present study, supporting our previous qualitative findings, we would conclude that the triangulation of evidence occurs. We used a two-pronged approach in Study 1: (a) generating Chinese OID statements for our initial item pool (i.e., the emic approach), and (b) reviewing the Western OID literature of commonly used measurement instruments for additional items (i.e., the etic approach). Last, we tested this item pool for scale reliability and validity using factor analytic procedures. We called our final measure the Chinese Organizational Identification Questionnaire (COIQ).

#### Hypothesis Testing for Measure Validation

To validate the COIQ, we hypothesized that the construct of Chinese OID as measured with the COIQ would be positively correlated with a couple of work-related constructs, namely workers’ self-concepts (*Hypothesis 1*) and overall job satisfaction (*Hypothesis 2*), because past research has shown that OID is positively linked with organizational members’ self-concepts (Dutton et al., 1994) and job satisfaction (Bartels et al., 2010; Başar and Basim, 2015). If our hypotheses are supported, those results would provide preliminary evidence of the criterion-related validity of the COIQ.

### Methods

#### Item Generation

We first followed Dillman et al. (2009) good item-writing principles and drafted an initial set of 39 Chinese OID items based on the interview results in our previous qualitative study (Yang and Nguyen, 2014). Secondly, we reviewed six commonly used measurement instruments of OID in the literature (i.e., Cheney, 1983; Mael and Ashforth, 1992; Doosje et al., 1995; Miller et al., 2000; Gautam et al., 2004; Edwards and Peccei, 2007) and identified 24 non-redundant items (e.g., covering cognitive, affective, motivational, and behavioral aspects of OID) to yield the initial emic-etic pool of 63 items. (In the interest of space, those items are available from the first authors upon request.)

#### Content Validity

Five business professors from the same institution with the first author voluntarily conducted a review of the content of this initial item pool. Specifically, they were asked to (a) assign an OID-based theme to each item in the pool, and (b) judge the items on concept relevance, clarity, simplicity, and ambiguity. Based on the ratings, items that were deemed too ambiguous, redundant, and/or not classifiable were dropped, supporting the content validity of the measure (see Messick, 1989). The result was a pool of 53 items (available upon request) which we used in subsequent analyses.

#### Participants

We first used *PowerSem 2* (Chan, 2009) to conduct an *a priori* power analysis for our CFA procedures. Based on an alpha of 0.05, the RMSEA 90% CI as the boundaries, and the medium-size power of 0.8, we found that we would need a sample of at least 137 participants to sufficiently test up to a three-factor model. Using the convenience sampling technique, we recruited Chinese workers to participate in our study by distributing 500 copies of the research questionnaire to MBA students who attended a large public university in central China. We asked those students to give the questionnaire and a written informed consent form (in accordance with the Declaration of Helsinki) to their network of full-time employed acquaintances and relatives in various enterprises across China. Participants in Study 1 were those who signed the written consent form and returned it along with the filled questionnaires in a stamped envelope to the researchers. This recruitment protocol in particular and the research study in general was approved by the Jiangxi University of Finance and Economics Academic Committee (which is the committee responsible for providing ethics approvals) and carried out in accordance with the recommendations of the University’s academic committee.

In total, *N* = 408 usable questionnaires were returned, constituting a response rate of 82% [greater than the typical response rate of 50% in Western behavioral science studies as noted by Baruch and Holtom (2008)].

Among those who declared their sex, there were 213 males and 196 females. (One participant did not declare their sex). The majority of participants (76%) were in the 20–25 age group. The two most common self-declared categories of jobs were operational staff (57%) and supervisors/managers (36.8%). The sample was educated: the majority of participants (70%) had a college or university degree or higher, whereas 18.6% were high school graduates. Participants were employed in a variety of company types (e.g., state-owned, private, foreign-invested, and corporations limited). A majority of participants (67.2%) belonged to companies with at least 1000 employees.

We utilized a random split-sample technique (Anastasi, 1988) to divide the overall sample into two sub-samples [see the description of the technique by Farrell and Rudd (2009) and Worthington and Whittaker (2006)]. We then conducted an exploratory factor analysis (EFA) with the first sub-sample (*n*_Study1-EFA_ = 203), followed by a series of confirmatory factor analyses (CFAs) with the second sub-sample (*n*_Study1-CFA_ = 205).

#### Missing Data

The missing data were replaced using total mean substitution (see Switzer et al., 1998). Although this method possibly leads to potential biased estimates, that issue was not probable in the present study because our missing data rates (7.39 and 7.77% for the two EFA and CFA subsamples, respectively) were smaller than the threshold criterion of 10% (Cohen and Cohen, 1983; Roth and Switzer, 1995). Also, although our follow-up Little’s missing-completely-at-random (MCAR) test showed a significant *p* value (*p* < 0.001), no identifiable pattern was found by using the *md.pattern* function in R. Alternatively, we reapplied FIML procedure to deal with missing values in the same data set and then reran the EFA but we found similar results and only negligible changes in the loadings of correlations.

#### Procedure

Participants were informed that the purpose of the questionnaire was for research only and participation was voluntary. The OID items were presented in a mixed order. No identification information was collected to ensure the anonymity of responses. After completing the questionnaire, participants mailed it back to the researchers in a provided stamped envelope addressed to the researchers.

#### Measures

Participants responded to the scale-development pool of 53 items of the COIQ using a 5-point Likert-type scale (ranging from “1 = strongly disagree” to “5 = strongly agree”). A composite scale score was computed by taking the average of item scores. Note that any measurement items originally written in English had been translated into Chinese and back to English, following Brislin’s (1976) rigorous six-step translation and back-translation process.

In our hypothesis testing, we used two single-item measures of the constructs of worker’s self-concept and overall job satisfaction: “Being a member of this organization is important to me” (Haslam et al., 1999) and “Overall, how satisfied are you with your job?” (Scarpello and Campbell, 1983). In the respective literatures, those single-item measures were found to be strongly correlated with other global measures of social and organizational identification (e.g., Mael, 1988; Doosje et al., 1995). Those measures were also positively correlated with multi-item measures of the respective constructs (e.g., *r*_c_ = 0.67 between Haslam et al.’s item and a multi-item measure of job satisfaction; Wanous et al., 1997).

#### Factor Analytic Strategy in Scale Development

We used the latest scale development practice of factor analyses advocated by Wegener and Fabrigar (2004) instead of following Nunnally and Bernstein’s (1994) classical test theory approach because Wegener and Fabrigar (2004) argue that the classical test theory may eliminate items that should not be eliminated or retain items that might weaken the construct validity of a measure.

### Results

#### Scale Development – EFA Findings

A principal axis factor analysis with direct oblimin rotation was carried out to allow for correlations between items of the COIQ (*n*_Study1-EFA_ = 203). Multiple factor retention rules were simultaneously applied in determining the factor retention (see Hu and Bentler, 1999; Tabachnick and Fidell, 2007). We also ran a parallel analysis (Watkins, 2000). Items were included in a factor if they displayed (a) a communality greater than 0.4, (b) a factor loading of at least 0.5, (c) a cross loading less than 0.32, (d) a cross-loading differential across factors greater than 0.15, and (e) if the items were conceptually coherent with other items on the factor (see Aquino and Lamertz, 2004). Therefore, the item deletion process was based on a rigorous procedure of factor analyses, and the remaining items reflect what Chinese participants believed about OID.

The final EFA iteration resulted in an 8-item, two-factor solution; the parallel analysis yielded similar results (see Table 1 for the factor loadings and Table 2 for item descriptive statistics). Note that none of the inter-item correlation magnitudes was greater than *r* = 0.604, meaning that the items were related but not the same.

**Table 1:** Factor loadings^a^ for exploratory factor analyses with direct oblimin rotation of the Chinese Organizational Identification Questionnaire (COIQ; Study 1, *n*_Study1-EFA_ = 203).

Item	Pattern Matrix	
	Factor 1	Factor 2
**“Emotion” Factor (5 items; α = 0.842)**		
1. I am glad I chose to work for my organization rather than another company.	**0.774**	–0.129
2. I would describe my organization as a large “family” to which most members feel a sense of belonging.	**0.756**	–0.010
3. I feel that my organization cares about me.	**0.704**	–0.020
4. I have a warm feeling toward my organization as a place to work.	**0.692**	0.080
5. I am proud to be an employee of my organization.	**0.649**	0.170
**“Behavior” Factor (3 items; α = 0.795)**		
6. I will maintain good interpersonal relationships in my organization.	0.002	**0.779**
7. I will actively strengthen the cooperation and communication with my colleagues.	–0.014	**0.772**
8. I am always willing to help my colleagues in need.	0.029	**0.709**

Eigenvalue	3.023	1.337
% Variance	37.784	16.710


*^a^Factor loadings > 0.40 are in boldface. The overall scale’s internal consistency was satisfactory: Cronbach’s α = 0.811.*

**Table 2:** Means, standard deviations and inter-correlations of the COIQ items (Study 1, *n*_Study1-EFA_ = 203).

Item	*M*	*SD*	1	2	3	4	5	6	7	8
1. I am glad I chose to work for my organization rather than another company.	3.13	1.06	–							
2. I would describe my organization as a large “family” to which most members feel a sense of belonging.	3.15	1.09	0.554**	–						
3. I feel that my organization cares about me.	2.99	1.10	0.524**	0.489**	–					
4. I have a warm feeling toward my organization as a place to work.	3.31	1.03	0.466**	0.561**	0.559**	–				
5. I am proud to be an employee of my organization.	3.42	0.91	0.537**	0.544**	0.453**	0.487**	–			
6. I will actively strengthen the cooperation and communication with my colleagues.	4.08	0.78	0.047	0.242**	0.134	0.252**	0.301**	–		
7. I will maintain good interpersonal relationships in my organization.	4.02	0.90	0.125	0.152*	0.164*	0.204**	0.258**	0.604**	–	
8. I am always willing to help my colleagues in need.	4.09	0.92	0.110	0.134	0.185**	0.241**	0.321**	0.542**	0.561**	–


*^∗^*p* < 0.05; ^∗∗^*p* < 0.01.*

The first factor (consisting of five items) represented 37.784% of the explained variance of the instrument. The content of those five items referred directly to one’s feeling about one’s organization, thus labeled “Emotional Attachment.” The second factor (three items) represented 16.710% of the explained variance. The item content reflected one’s efforts on maintaining interpersonal harmony and solidarity within one’s organization through behaviors such as interaction and cooperation, thus labeled as “Striving for Solidarity and Cooperation.” Note that the “emotion” items came from the Western item pool, whereas the “behavior” items came from the Chinese item pool.

#### Scale Dimensions: CFA Findings

The reliability and the dimensionality of the 8-item COIQ were assessed using the CFA subsample (*n*_Study1-CFA_ = 205) with maximum likelihood (MLE) estimation method in Amos 22.0 (Arbuckl, 2013). Specifically, following Bollen and Long’s (1993) procedure, we tested *Model 1* in which the eight indicators loaded onto their intended two factors according to the EFA final solution. The two factors were allowed to covary freely. Some research has shown that the construct of OID is uni-dimensional (e.g., Ashforth and Mael, 1989; Gautam et al., 2005); therefore, we explored the fit of a competing one-factor model (*Model 2*). Based on Cheung and Rensvold’s (2002) recommendation, we obtained the following fit indices for the competing models: Chi-square, Standardized Root Mean Square Residuals (SRMSR), Comparative Fit Index (CFI), and Tucker-Lewis Index (TLI).

**FIGURE 1:**
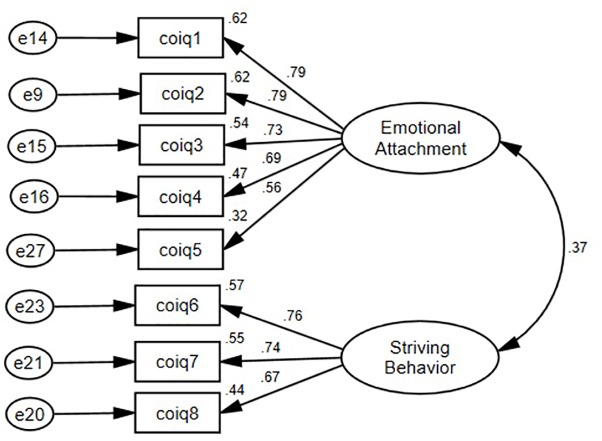
Model specification of the 8-item, bi-dimensional Chinese Organizational Identification Questionnaire (Study l).

Table 3 presents the model fit indices of the two models, whereas Table 4 shows the loadings of the bi-dimensional model. As shown, the 8-item, bi-dimensional model (*Model 1*) fit the Chinese data the best, yielding good fit indices (see Figure 1). Also, the standardized loadings of the indicators ranged from 0.564 to 0.790 and were statistically significant. We thus concluded that the bi-dimensional, correlated model of Chinese OID adequately described the pattern of relationships among the eight items.

**Table 3:** The fit indices for competing confirmatory factor analytic models of the COIQ (Study 1, *n*_Study1-CFA_ = 205).

Model	χ^2^	*df*	χ^2^/*df*	*p*	RMSEA (90%CI)	CFI	TLI	PNFI	CAIC	ECVI
1	20.535	19	1.081	0.363	0.020 (0.000, 0.066)	0.997	0.996	0.654	128.027	0.267
2	139.687	14	9.978	<0.001	0.210 (0.179, 0.242)	0.685	0.527	0.445	228.210	0.822


*Model 1 = The 2-factor correlated model (the best-fit model); Model 2 = The one-factor model.*

**Table 4:** Confirmatory factor analytic findings of the bi-dimension correlated model of the COIQ (Study 1, *n*_Study1-CFA_ = 205).

Dimension	Item	Standardized Loading	SMC	AVE	SIC*	CR
“Emotional Attachment” (Affective component, 5 items)	1	0.790	0.624	0.514	0.137	0.839
	2	0.786	0.618			
	3	0.733	0.538			
	4	0.689	0.475			
	5	0.564	0.318			
“Striving for Solidarity and Cooperation” (Behavioral component, 3 items)	6	0.757	0.572	0.524	0.137	0.767
	7	0.745	0.555			
	8	0.667	0.445			


*^∗^SIC = Square inter-construct correlations.*

#### Criterion-Related Validity

We tested the criterion-related validity of the COIQ measure using the CFA sub-sample. We hypothesized that there were positive relationships between the COIQ dimensions and participants’ organizational self-concepts (*Hypothesis 1*), and between the dimensions and participants’ overall job satisfaction (*Hypothesis 2*). Both hypotheses were supported: the two dimensions of the COIQ were significantly correlated (at *p* < 0.01) with organizational self-concepts: *r* = 0.246 for “Emotional Attachment,” and *r* = 0.448 for “Striving for Solidarity and Cooperation.” There were also significant relationships between the COIQ dimensions with overall job satisfaction (at *p* < 0.01): *r* = 0.708 for “Emotional Attachment,” and *r* = 0.243 for “Striving for Solidarity and Cooperation.” The overall COIQ scale score was also positively related to organizational self-concepts (*r* = 0.376, *p* < 0.01) and overall job satisfaction (*r* = 0.666, *p* < 0.01). Taken together, our results provided the preliminary evidence of the COIQ’s criterion-related validity.

Additionally, we assessed the convergent and discriminant validity of the COIQ by analyzing the AVE (Fornell and Larcker, 1981), and the relationship between the AVE estimates and corresponding SIC, or correlations between each pair of latent variables (see Fornell and Larcker, 1981; Hair et al., 1995). The AVE of each construct should be above 0.50 (Bagozzi and Yi, 1988) to support the existence of convergent validity of our constructed COIQ. If the value of AVE of each latent variable is substantially higher than that of SIC, discriminant validity would exist. The results supported our expectation: all AVE estimates were greater than the corresponding SICs, meaning that the indicators had more in common with the construct with which they were associated than with other constructs, yielding further convergent validity evidence for the COIQ.

#### Testing for Common Method Bias

We tested for possible common method bias in the data in two ways: First, we applied Harman’s single-factor test (cf. Podsakoff and Organ, 1986) to evaluate the amount of variance in observed variables that can be explained by a single factor. We found that the single factor only accounts for 43.119% of the variance in our study, whereas the amount of variance less than 50% is desirable according to Podsakoff and Organ (1986). Secondly, we compared the standardized regression weight differences of each path between *Model 1* and *Model 2* with one latent common-method factor (see Gaskin, 2016). We found that the differences between the two models only ranged from 0.053 (Item 3) to 0.245 (Item 8). Taken together, the two tests indicated that it was possible but not probable that there was significant common method bias in the COIQ data.

### Discussion

In Study 1, following Cheung et al.’s (2001) emic-etic approach, we developed a measure of Chinese perception of OID, called the COIQ. To the best of our knowledge, this is the first emic-etic measure of Chinese OID in the culture-specific literature. Our contribution is that we took into account both the universality of OID in the existing (Western) literature and the culture-specific OID interpretation of Chinese workers. Our results were partly consistent with past research: (a) the affective component is a core element of OID (see Johnson and Morgeson, 2005), and (b) the OID concept refers to the perception of unity with or belonging to an organization (see Ashforth and Mael, 1989). However, the uniqueness of our findings is that Chinese employees’ idea of unity with an organization is a *guanxi*-oriented behavioral tendency, not just a cognitive perception. The factor structure from the COIQ measurement was thus consistent with our previous qualitative support for the bi-dimensional model of the Chinese OID concept.

The theoretical implications of our study to the broad OID literature are that (a) the affective dimension of Chinese OID might predict one’s expectations of organizational support (J. W. Jackson, 1999), and that (b) the more employees emotionally identify with their organizations, the more likely they might internalize their organization’s desired emotions (see Mishra and Bhatnagar, 2010). The practical implication is that the more intrinsically motivated Chinese employees are, the more likely they would behave in a manner consistent with their organization interests, and the greater their OID level with the organization is (see van Knippenberg and Sleebos, 2006).

#### Limitations

Because we collected cross-sectional data in the present study, it was possible that common method bias might have influenced our findings. However, we used two appropriate statistical procedures to test for any common method bias effects and the results of both tests were not statistically significant. In other words, the effect of common method bias on our findings was possible but not probable. Another limitation is the use of single-item measures in testing for the relationships of Chinese OID and other variables.

The fact that the majority of our employee participants were young adults (between 20 and 25 years old) might also be considered a limitation of our study. However, the composition of our sample might have reflected that of the actual workforce and/or the demographic trend in China. Not only in Asia but also around the world, an increasingly substantial portion of the workforce consists of Millennials who may have different expectations of their organization (e.g., Ng et al., 2010), influencing the interpretation of OID concept. Nevertheless, age or generation differences in OID might be of a concern and should be addressed in a future study.

## Study 2 – Cross-Validating the COIQ and Mapping Out Chinese OID Profiles

### Introduction

As aforementioned, we followed Messick’s (1989) best practices for establishing a measure’s construct validity by searching for patterns of relationships among the COIQ and other organizational measures across samples. Therefore, in Study 2, we proceeded with examining three research questions: First, would the bi-dimensional structure of the COIQ (i.e., the Chinese OID construct itself) prevail when being tested with a different sample of job incumbents? Secondly, how would the Chinese OID construct be related to other organizational antecedents and/or outcomes? Positive findings for those two questions would further support the Chinese OID’s construct validity. More importantly, given the aforementioned talent retention crisis and the management context in Chinese organizations, we explored the third, practical research question: Could Chinese employers use the COIQ to generate profiles of firm identifiers among their workers? Therefore, we developed a series of hypotheses testing our research questions in the present study.

#### Bi-Dimensional OID

In *Hypothesis 1*, we hypothesized that the Chinese OID concept would emerge as two related but distinct dimensions, consistent with Study 1 findings.

#### Link to Psychological Contract

According to Ashforth and Mael (1989), OID depicts the sensing of “unity” with an organization and is rooted in the framework of Social Personality Theory (SPT; Hogg and Terry, 2000). However, when employees perceive that certain psychological contracts with their organization have been violated, employees would experience an inconsistency regarding their organizational membership. As the result, employees would start engaging in a process of distancing themselves from their organization, consequently reducing their level of OID (Norton et al., 2003; Lane and Scott, 2007).

Therefore, in *Hypothesis 2*, we predicted that the Chinese OID dimensions would be negatively predicted by workers’ perceived *psychological contract violation* (PCV; Robinson and Morrison, 2000) which assesses employees’ feelings of disappointment arisen from their belief about how their organization fails to fulfill its work-related promises.

#### Links to Perceptions of Compensation and Benefit Practices

The two constructs of perceived compensation practices (PCP; Huang, 2002) and perceived benefits practices (PBP; I.P. Chen, 2003) were chosen to test the convergent validity of the COIQ dimensions because they would conceptually predict one’s OID level: the more favorably a worker perceived their company’s practices of worker compensation and/or benefits, the more strongly that they would identify with their organization.

Therefore, in *Hypothesis 3* and *Hypothesis 4*, we expected that Chinese workers’ perceptions of compensation practices and benefit practices, respectively, would positively predict Chinese workers’ OID dimensions.

#### Link to Unethical Pro-organizational Behavior

The construct of unethical pro-organizational behavior (UPB; Umphress et al., 2010) is defined as unethical behaviors conducted by an employee to benefit the organization. In the present study, we predicted that one’s OID level would provide the social context for how one would behave (ethically or not) based on the social identity theory (see Hogg et al., 1995); specifically, employees might behave in ways to maintain or enhance a positive self-image of being affiliated with their organization (i.e., behaving ethically).

Therefore, in *Hypothesis 5*, we predicted that the Chinese OID dimensions would negatively predict workers’ *UPB*.

### Methods

#### Participants

Upon our request, the human resources departments of 13 companies across China randomly distributed 540 questionnaires along with written consent forms and a stamped envelope addressed to the researchers to workers in their organizations. The companies are located in Central China (Jiangxi, Anhui), Southwest China (Sichuan), Eastern China (Jiangshu), Northern China (Beijing), and Southern China (Guangdong), thus representing a diverse body of Chinese workers. Participants were explained by HR staff about the nature of our study before filling out the questionnaire; they were also informed that valid surveys would be entered in a drawing contest to win an I-pad. Those who were interested in participating in the study signed the written consent form (in accordance with the Declaration of Helsinki) and returned their completed survey and consent form in a sealed envelope to the HR contacts of the researchers, who then forwarded the sealed envelopes to the researchers themselves. This recruitment protocol in particular and the research study in general was approved by the Jiangxi University of Finance and Economics academic committee and the present study was carried out in accordance with the committee’s recommendations.

After 4 weeks, a majority of respondents (*N* = 408) returned their questionnaires (a 75.93% response rate). Using Gaskin’s (2016) suggested data screening procedure, the authors found *N* = 299 valid questionnaires to be included in the subsequent data analyses (a high valid response rate of 55.37%). Participants’ average age was 32.37 years (*SD* = 8.54), and 50.2% were male. In terms of work tenure, 37.1% of participants had worked for 2–5 years in their current organization.

#### Measures and Procedure

The study was part of a larger study examining human resource management practices and their effects in organizations. In addition to the 8-item COIQ scale (see Table 2 above), the researchers administered four relevant organizational measures to participants. The first measure was the 6-item, 7-point Likert scale of *PCP* based on Huang’s (2002) study, with a satisfactory internal consistency in the present study (Cronbach’s α = 0.84). A sample item is “Incentive pay system, such as profit sharing, stock option or bonus system, is offered to encourage employees to pursue organizational goals.”

The second measure is the 9-item, 7-point Likert scale of *perceived* *benefit practices* (PBP) based on Chen’s (2003) work, with excellent internal consistency (Cronbach’s α = 0.94). A sample item is “The care service for the elderly/children in the home is provided [by the company].”

The third measure is the 6-item, 5-point Likert scale assessing *UPB* (Umphress et al., 2010) with satisfactory internal consistency (Cronbach’s α = 0.85). A sample item is “If it would help my organization, I would misrepresent the truth to make my organization look good.”

The fourth measure is the 4-item, 5-point Likert scale that measures *PCV* (Robinson and Morrison, 2000), with excellent internal consistency (Cronbach’s α = 0.92). A sample item is “I feel betrayed by the organization.”

#### Factor Analyses

We used the confirmation factor analytic strategy similar to that in Study 1 to cross-validate the COIQ measure in the present study.

#### Latent Profile Analyses

To determine whether meaningful latent profiles of Chinese employees could be identified on the basis of their levels of OID across two COIQ dimensions, we conducted a series of latent profile analyses (LPA; Gibson, 1959; Lazarsfeld and Henry, 1968; Bartholomew, 1987; Muthén, 2002). LPA is a model-based approach to systematically analyze meaningful relationships in subgroups of individuals, clustering them into qualitatively and quantitatively distinct patterns or profiles (Morin et al., 2011). Because the current study aimed at identifying employee OID profiles rather than to verify the invariance of the COIQ measurement across profiles, all LPA models were specified with a class-invariant factor model in which only the indicators’ intercepts were allowed to vary across classes (Lubke and Muthén, 2005).

A series of LPA was conducted to explore the profile membership via the expectation–maximization algorithm of the robust maximum likelihood estimator (MLR; Muthén and Shedden, 1999). We followed Ram and Grimm’s (2009) suggested flowchart to identify the best candidate model. The first step is examining the output of each model estimates for potential problems. Second, models with different numbers of classes are compared using information criteria (IC-based) fit statistics, including the Akaike Information Criterion (AIC; Akaike, 1987), the Bayesian Information Criterion (BIC; Schwarz, 1978), and the Sample-Adjusted BIC (SABIC; Yang, 2006) based on L^2^. The smaller the fit statistics is, the better fit is the model to the data.

Third, the classification accuracy of a latent profile model was examined. As a measure of aggregated classification uncertainty, entropy values were assessed (see Ramaswamy et al., 1993). In previous research, entropy values higher than 0.80 have been viewed as a good classification indicator, meaning that the latent profiles are highly discriminating, and entropy values higher than 0.60 have been viewed as acceptable (see Muthén, 2004). In addition, we also examined the models with number of groups that had less than 1% and less than 5% of the cases because solutions with small number of cases may not be feasible (see Marsh et al., 2009).

Fourth, the statistical model comparison likelihood ratio tests and bootstrapping procedures were used to avoid the sensitivity of the indicators to sample size. Significant *p*-values (*p* < 0.05) associated with the likelihood ratio test would support the retention of a more complex solution with at least *k* profiles. In other words, if the *p* value is less than 0.05, the *k* model is preferable; otherwise, the *k* - 1 model is a better fit for the data. An additional consideration is the size of the smallest class. Lubke and Neale (2006) proposed that researchers must be able to defend what is gained by the addition of one class if the additional class is composed of a relatively small number of members (i.e., proportionally less than 1.0% and/or numerically *n* < 25).

In LPA, the final parameter estimates chosen are those associated with the highest likelihood value. As there is no clear-cut rule for the best log-likelihood value, we followed Morin et al. (2011) suggestion to re-run a related model with multiple random sets of start values for at least five times to check that the best log-likelihood was still obtained and replicated. If the addition of one class led to a suboptimal solution (i.e., a false maximum likelihood), we would not proceed further.

The process began with a 2-class model, then we increased the number of classes by one in each subsequent iteration until non-convergence occurred (see Muthén and Muthén, 1998–2015; Lubke and Muthén, 2005; Pastor et al., 2007). Then Ram and Grimm’s (2009) four-step process was applied to analyze the data. The algorithm used was integration and the number of initial stage random started at 800 with 40 best sets of start values being chosen for final stage optimization, so as to avoid the inadequate start value leading to the convergence on a local solution (i.e., false maximum likelihood; see McLachlan and Peel, 2000; Hipp and Bauer, 2006).

The number of initial stage iterations of iterations was 40 and the estimator used was MLR. To get the *p*-value of aLMR-LRT and BLRT results by TECH 11 and TECH 14 options (see Asparouhov and Muthén, 2012), we set the LRTSTARTS at 10 5 50 20 so as to give more random starts for the default sequential approach, while the default setting of LRTBOOTSTRAP (100) was adopted to request the number of bootstrap draws for non-sequential approach, so as to reduce the computation time. Missing data were handled using the MLR estimator (maximum likelihood estimation with robust standard errors). Moreover, within-class means and variances of the observed variables were estimated, with residual covariances between the indicators fixed to zero, consistent with the assumption of local independence in classical LPA that indicators within groups are uncorrelated and associations among indicators are explained in terms of the grouping variables (see Bartholomew, 1987; Uebersax, 1999; McLachlan and Peel, 2000; Muthén, 2001). The obtained solution would be clearly replicated and the log-likelihood value would also be replicated many times. Thus, it could be assumed that these solutions represented the best fit solution.

### Results

#### Cross-Validation – CFA Findings

**FIGURE 2:**
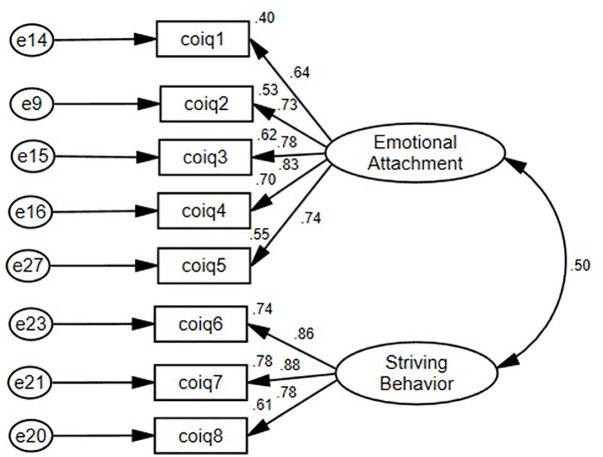
Model specification of the cross-validated, 8-item, bi-dimensional Chinese Organizational Identification Questionnaire (Study 2).

To test *Hypothesis 1*, predicting that the Chinese OID construct would emerge as a bi-dimensional one from the COIQ data, we conducted a series of CFAs using Amos 22 (Arbuckl, 2013), testing two competing models to see which would fit the COIQ data better. Table 5 summarizes the fit indices of those two models, whereas Figure 2 shows the standardized item loadings on the two-factor model. As expected, the two-factor correlated model fit the data better than the one-factor model, which was consistent with Study 1 results. Therefore, *Hypothesis 1* was supported. Note that the inter-correlation between the two COIQ dimensions was of a positive and moderate magnitude, *r* = 0.50, indicating that the two COIQ dimensions have a reasonable overlap but they are still two distinct sub-constructs as expected.

**Table 5:** The fit indices for two competing confirmatory factor analytic models of the COIQ (testing *Hypothesis 1*, Study 2, *N* = 299).

Model	χ^2^	*df*	χ^2^/*df*	*p*-value	RMSEA (90%CI)	CFI	TLI	PNFI	CAIC	ECVI
1	39.087	19	2.057	0.004	0.06 (0.032, 0.086)	0.983	0.975	0.657	0.667	0.299
2	388.904	20	19.445	0.000	0.249 (0.228, 0.271)	0.690	0.566	0.486	–	1.466


*Model 1 = The bi-dimensional correlated model (the best-fit model); Model 2 = The one-factor model.*

#### Hypothesis Testing – Regression Findings

To test our hypotheses regarding the COIQ’s various validity indices, we conducted a series of bivariate correlation analyses (Table 6) and a series of linear regression analyses (Tables 7, 8), examining the possible relationships between the two COIQ dimensions and other work-related antecedents and/or outcomes of Chinese OID.

**Table 6:** Descriptive statistics of and bivariate correlations between the COIQ dimensions and the antecedents and the outcome variables (Study 2).

Variable	*M*	*SD*	1	2	3	4	5	6	7
1. COIQ – Affect (Emotional Attachment)	4.98	1.15	(0.86)						
2. COIQ – Behavior (Striving for Solidarity and Cooperation)	5.82	1.03	0.43**	(0.88)					
3. Perceived Compensation Practices	4.63	1.37	0.43**	0.32**	(0.84)				
4. Perceived Benefits Practices	4.33	1.50	0.39**	0.18**	0.76**	(0.94)			
5. Perceived Psychological Contract Violation	2.33	0.93	–0.43**	–0.49**	–0.19**	–0.03	(0.92)		
6. Unethical Pro-Organizational Behavior	2.54	0.78	0.15**	–0.13*	0.15*	0.19**	0.35**	(0.85)	


*Diagonal values are the scale Cronbach’s α coefficients. ^∗^*p* < 0.05; ^∗∗^*p* < 0.01.*

**Table 7:** Hypothesis testing: predictors of the COIQ dimensions (Study 2).

Variable	*B*	*S.E.*	*Beta*	95% CI
*Hypothesis 2a: Predicting COIQ Emotional Attachment*				
Constant	6.223	0.165		(5.898, 6.547)
**Psychological contract violation**	–0.533**	0.066	–0.429	(–0.663, –0.404)
*R*^2^	0.184			
*F*(1, 292)	65.831**			

*Hypothesis 2b: Predicting COIQ Striving Behavior for Solidarity and Cooperation*				
Constant	7.097	0.142		(6.817, 7.377)
**Psychological contract violation**	–0.545**	0.057	–0.490	(–0.657, –0.433)
*R*^2^	0.241			
*F*(1, 292)	92.469**			

*Hypothesis 3a: Predicting COIQ Emotional Attachment*				
Constant	3.171	0.211		(2.755, 3.587)
**Perceived compensation practices**	0.393**	0.044	0.460	(0.306, 0.479)
*R*^2^	0.212			
*F*(1, 295)	83.318**			

*Hypothesis 3b: Predicting COIQ Striving Behavior for Solidarity and Cooperation*				
Constant	4.727	0.202		(4.329, 5.126)
**Perceived compensation practices**	0.238**	0.042	0.311	(0.155, 0.321)
*R*^2^	0.097			
*F*(1, 295)	31.700**			

*Hypothesis 4a: Predicting COIQ Emotional Attachment*				
*Constant*	3.691	0.193		(3.311, 4.070)
**Perceived benefit practices**	0.300**	0.042	0.389	(0.217, 0.383)
*R*^2^	0.152			
*F*(1, 284)	50.732**			

*Hypothesis 4b: Predicting COIQ Striving Behavior for Solidarity and Cooperation*				
Constant	5.259	0.185		(4.894, 5.623)
**Perceived benefit practices**	0.127**	0.040	0.183	(0.048, 0.207)
*R*^2^	0.034			
*F*(1, 284)	9.886**			


*CI = confidence interval. ^∗^*p* < 0.05; ^∗∗^*p* < 0.01.*

**Table 8:** The COIQ dimensions predicting unethical pro-organizational behavior (*Hypothesis 5*, Study 2).

Variable	*B*	*S.E.*	*Beta*	95% CI
Constant	2.721	0.271		(2.188, 3.253)
COIQ – Emotional attachment	0.170**	0.042	0.253	(0.086, 0.253)
COIQ – Striving behaviors	–0.176**	0.048	–0.234	(–0.269, –0.082)
*R*^2^	0.068			
*F*(2, 285)	10.427**			


*CI = confidence interval. ^∗^*p* < 0.05; ^∗∗^*p* < 0.01.*

*Hypothesis 2* was supported. Both COIQ dimensions (emotion and behavior) were significantly predicted by respondents’ perceived psychological contract breach in the hypothesized inverted direction. The more workers felt disappointed in their company because of perceived betrayal, the less they felt attached to their organizational identity, and the less likely they would act in favor of the company’s goals.

Both *Hypotheses 3* and *4* were supported. Regressing the perceived business practices of compensation and benefits onto the two COIQ dimensions, respectively, we found that favorable perceptions of those company practices positively predicted workers’ higher OID level on both dimensions.

*Hypothesis 5* was supported, but interestingly, only the behavioral component of the COIQ negatively predicted respondents’ UPB, whereas the link between the COIQ emotional dimension and UPB was opposite to the hypothesized direction. As expected, the more inclined workers felt to stand with their organization and act toward their organization’s goals, the less likely they wanted to do anything unethical, even though the unethical act was supposedly for the organization’s benefit. However, the opposite finding was true when the affective component of Chinese OID became the predictor of one’s UPB: the more one felt emotionally attached to their company, the more likely they would act unethically for the good of their company.

#### Hypothesis Testing – Structural Equation Modeling Findings

**FIGURE 3:**
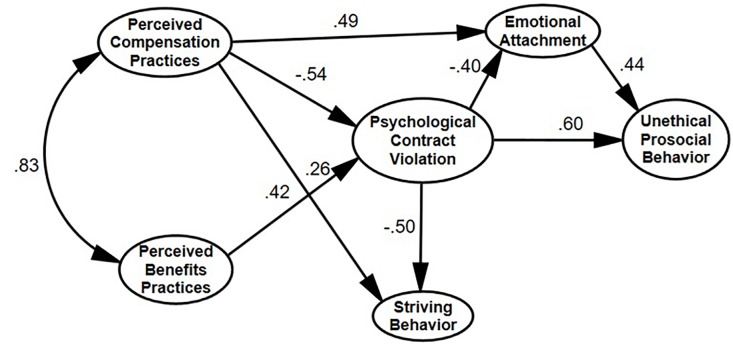
Hypothesis model testing: the structural equation modeling results (Study 2). Note that Emotional Attachment and Striving Behavior are the dimensions of the Chinese Organizational Identification Questionnaire.

In addition to the linear regression analyses, we ran a structural equation modeling (SEM) analysis, re-testing Hypotheses 2–5 by including both COIQ dimensions and their organizational antecedents and outcome variables into a conceptual model as an exploration of the whole network of relationships. Figure 3 displays the magnitudes and directions of the inter-variable relationships.

As expected, the SEM findings showed that the model had a good fit to the data: χ^2^ = 1197.046, *df = 455*, χ^2^/*df* = 2.631; CFI = 0.885; TLI = 0.866, RMSEA = 0.074 [0.069, 0.079]. Those results were similar to the linear regression findings. Interestingly, we found that the PBPs had no direct influence over the COIQ dimensions, whereas the PCPs significantly predicted both dimensions of the COIQ. Those findings further substantiated the divergence of the bi-dimensional structure of Chinese OID. Note that when we added several possible control variables of gender, age, work experience and education into this SEM model, the resulting model fit indices were slightly different from those in the original model (without the control variables), but those differences were negligible: χ^2^ = 1391.948, *df = 575*,χ^2^/*df* = 2.421; CFI = 0.879; TLI = 0.859, RMSEA = 0.069 [0.064, 0.074].

#### Latent Profile Analytic Results – Three OID Profiles

In terms of the practical HR usage of the COIQ, we were interested in how the COIQ measure could be used to classify individuals regarding their tendency of identifying with their organizations based on their scores on both COIQ dimensions. Using M-Plus software (Muthén and Muthén, 2007), we conducted a series of LPAs, following McLachlan and Peel’s (2000) suggestions of appropriate statistical tests and indices to determine an optimal number of profiles to retain.

**FIGURE 4:**
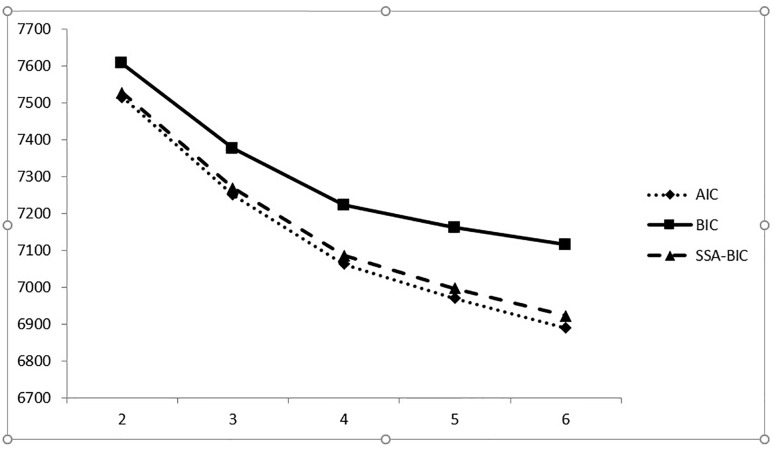
The Elbow Plot of the information criteria for the latent profile analysis without covariates.

For the three information indexes (AIC, BIC, and SABIC), their values continued to decrease across the range of models considered, but only marginally so between the four-class solution and the six-class one. The comparison of the AIC, BIC, and SABIC values for the six different models was done using an elbow plot (see Figure 4 that shows the point of formation of a first angle), which suggested that the improvement in fit reached a plateau at the forth profile and became negligible thereafter. The adjusted Lo et al. (2001) likelihood ratio test (aLMR) became non-significant with the four-class model, meaning that the three-class model was optimal.

**FIGURE 5:**
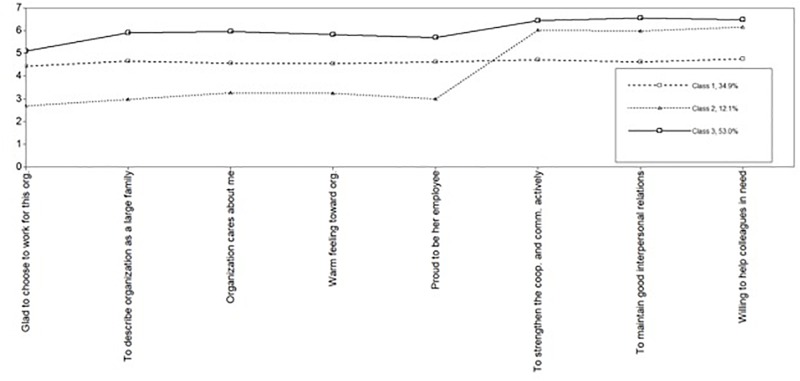
Results of a latent profile analysis, showing the classification of organizational identifiers as strong identifiers, moderate identifiers and action-oriented identifiers.

The entropy values were higher than the suggested 0.80 value and almost identical among the three-class, four-class, five-class, and six-class solutions. High posterior probabilities (in the 90% range) indicated that there was a high confidence that an individual assigned to a given class actually belonged to that class. Based on all of those considerations, we concluded that a three-profile solution provided the best fit indices to the COIQ data (see Table 9 for the fit indices and Figure 5 for the LPA map result).

**Table 9:** Model fit indices for two- to six-class solutions of Chinese Organizational Identification without covariates.

Model (Class Selection)	LL	Best H0 replicated	FP	AIC	BIC	SSA-BIC	aLMR-LRT(p)	Entropy
LPA models without Covariates								
Two classes	–3732.499	YES	25	7514.997	7607.508	7528.224	<0.001	0.839
**Three classes**	–**3591.485**	**YES**	**34**	**7250.970**	**7376.785**	**7268.958**	**0.032**	**0.894**
Four classes	–3488.576	YES	43	7063.153	7222.272	7085.902	0.148	0.878
Five classes	–3432.671	YES	52	6969.343	7161.766	6996.854	0.226	0.877
Six classes	–3383.867	YES	61	6889.734	7115.461	6922.006	0.077	0.900


*Bold indicates the best-fit model.*

In other words, in our sample, there appeared to be three OID profiles of Chinese workers: (a) those who strongly identified with their company both emotionally and in their behaviors (thus labeled as the “Strong Identifier” group); (b) those who moderately identified with their organization on both dimensions (thus labeled as the “Moderate Identifier” group), and (c) those who declared that they were not emotionally connected with their organization, but were paradoxically committed to pro-organization actions as much as the strong identifiers (thus labeled as the “Action-Oriented Identifier” group). Table 10 presents the mean scores for each of the Chinese OID variables across the three profiles.

**Table 10:** COIQ scale means across the three-profile solution.

	Profile 1: “Action-Oriented Identifiers” *n* = 35 (11.706%)		Profile 2: “Moderate Identifiers” *n* = 106 (35.452%)		Profile 3: “Strong Identifiers” *n* = 158 (52.843%)	
	*M*	*SD*	*M*	*SD*	*M*	*SD*
Factor 1: *Emotional Attachment*	2.994	1.023	4.559	0.621	5.704	0.713
Factor 2: *Striving Behavior*	6.076	0.662	4.701	0.691	6.508	0.510


*The classification posterior probabilities for Profiles 1–3 are 94.9, 93.2, and 96.5%, respectively, meaning that the probability for individuals belonging to their respective classified profile was very high.*

#### Mean Differences Among the OID Profiles

Although not hypothesized, we further explored possible mean discrepancies of relevant organizational variables of interest among the three OID profiles. Conducting a series of One-Way ANOVAs and the corresponding *post hoc* tests wherever appropriate, we found that there were significant group differences in psychological contract violation (PCV), perceived compensation and benefit practices (PCP and PBP), and the unethical pro-organization behavior variable (UPB). (See Tables 11, 12 for group descriptive information and *F*-values.)

**Table 11:** Organizational Variable Mean Differences across the Three OID Profiles.

Variable	OID Profile	*N*	*M*	*SD*	*SE*
PCV	Level 1 (Moderate Identifiers)	104	2.82	0.65	0.06
	Level 2 (Strong Identifiers)	155	1.93	0.91	0.07
	Level 3 (Action-Oriented Identifiers)	35	2.67	0.87	0.15
UPB	Level 1 (Moderate Identifiers)	104	2.69	0.69	0.07
	Level 2 (Strong Identifiers)	150	2.53	0.83	0.07
	Level 3 (Action-Oriented Identifiers)	34	2.15	0.70	0.12
PCP	Level 1 (Moderate Identifiers)	105	4.26	1.01	0.10
	Level 2 (Strong Identifiers)	157	5.06	1.28	0.10
	Level 3 (Action-Oriented Identifiers)	35	3.48	1.61	0.27
PBP	Level 1 (Moderate Identifiers)	103	4.22	1.18	0.12
	Level 2 (Strong Identifiers)	153	4.65	1.49	0.12
	Level 3 (Action-Oriented Identifiers)	30	3.12	1.83	0.33


*PVC = psychological contract violation; UPB = unethical pro-organizational behavior; PCP = perceived compensation practices; PBP = perceived benefit practices.*

**Table 12:** ANOVA tables – the three OID profiles.

Variable		*SS*	*df*	*MSE*	*F*	*p*
PVC	Between Groups	54.631	2	27.315	40.385	0.000
	Within Groups	196.827	291	0.676		
	Total	251.458	293			
UPB	Between Groups	7.594	2	3.797	6.459	0.002
	Within Groups	167.524	285	0.588		
	Total	175.118	287			
PCP	Between Groups	89.640	2	44.820	29.215	0.000
	Within Groups	451.033	294	1.534		
	Total	540.673	296			
PBP	Between Groups	60.866	2	30.433	14.947	0.000
	Within Groups	576.219	283	2.036		
	Total	637.086	285			


*PVC = psychological contract violation; UPB = unethical pro-organizational behavior; PCP = perceived compensation practices; PBP = perceived benefit practices.*

Subsequent *post hoc* analyses (Table 13) showed that for the PVC, the Strong Identifiers significantly perceived much less PVC than the other two groups, but there was no significant difference on PVC between the Moderate Identifiers and the Action-Oriented Identifiers. In terms of UPB, the Action-Oriented Identifiers were the least likely to engage in such behavior among the three groups, but there was no significant difference between the Strong Identifiers and the Moderate ones. In terms of PCP, all three group means were significantly different from one another, with the Strong Identifiers perceived their company’s compensation the most favorably, followed by the Moderate Identifiers and the Action-Oriented Identifiers. Regarding employees’ perceptions of their firm benefits, both the Strong Identifiers and the Moderate Identifiers had significantly more favorable perceptions than the Action-Oriented Identifiers, but those two groups did not differ significantly from each other.

**Table 13:** Scheffe *post hoc* tests of mean differences amongst the three OID profiles.

Variable	(I) Profile	(J) Profile	Mean Difference (I-J)	*SE*	*p*	95% Confidence Interval	
						Lower	Upper
PCV	1	2	0.897*	0.104	0.000	0.641	1.154
		3	0.153	0.161	0.636	–0.242	0.549
	2	1	–0.897*	0.104	0.000	–1.154	–0.641
		3	–0.744*	0.154	0.000	–1.123	–0.365
	3	1	–0.153	0.161	0.636	–0.549	0.242
		2	0.744*	0.154	0.000	0.365	1.123
UPB	1	2	0.160	0.098	0.263	–0.081	0.401
		3	0.543*	0.152	0.002	0.17	0.915
	2	1	–0.160	0.098	0.263	–0.401	0.081
		3	0.382*	0.146	0.033	0.024	0.741
	3	1	–0.543*	0.152	0.002	–0.915	–0.17
		2	–0.382*	0.146	0.033	–0.741	–0.024
PCP	1	2	–0.800*	0.156	0.000	–1.184	–0.416
		3	0.785*	0.242	0.006	0.190	1.38
	2	1	0.800*	0.156	0.000	0.416	1.184
		3	1.585*	0.232	0.000	1.015	2.155
	3	1	–0.786*	0.242	0.006	–1.380	–0.190
		2	–1.586*	0.232	0.000	–2.155	–1.015
PBP	1	2	–0.426	0.182	0.065	–0.875	0.020
		3	1.1036*	0.296	0.001	0.375	1.832
	2	1	0.428	0.182	0.065	–0.020	0.875
		3	1.531*	0.285	0.000	0.830	2.232
	3	1	–1.103*	0.296	0.001	–1.832	–0.375
		2	–1.531*	0.285	0.000	–2.232	–0.830


*PVC = psychological contract violation; UPB = unethical pro-organizational behavior; PCP = perceived compensation practices; PBP = perceived benefit practices. Profile: (1) Moderate Identifiers; (2) Strong Identifiers; (3) Action-Oriented Identifiers. (^∗^) The mean difference is significant at the 0.05 level.*

### Discussion

#### Chinese OID Construct Validity

In this cross-validation study, we found further support for the construct validity of COIQ: the bi-dimensional structure of Chinese OID prevailed, consistent with the qualitative results in our unpublished study (Yang and Nguyen, 2014) and the quantitative findings from Study 1. Moreover, most of our hypotheses regarding the validity of the COIQ were supported, shedding further light on how valid our Chinese OID construct was in the context of other organizational antecedents and outcomes.

A fascinating finding is that the two COIQ dimensions diverged in terms of predicting workers’ unethical pro-organization behavior. As expected, we found a negative relationship between the COIQ behavioral sub-scale with the UPB, consistent with the findings in Umphress et al. (2010). However, we also found that, when workers declared a greater level of emotional attachment to their organization, they surprisingly became more likely to engage in unethical behaviors for the good of the company. Although in the opposite direction of our hypothesis, this finding was consistent with Ashforth and Anand’s (2003) research, finding that those who strongly identified themselves with their organization would choose to disregard personal moral standards and engage in acts that favored the organization, even at the expense of those outside their organization. Recently, Chen et al. (2016) were able to replicate that particular relationship between OID and UPB in both Chinese and US samples. Nevertheless, our contribution to the literature on OID-UPB linkage is that our divergent findings have shed further light onto the seemingly contradicting results in the previous literature: perhaps the aforementioned research has inadvertently tapped onto two different aspects of OID, hence activating differential UPB reactions, which should be further examined in future research.

Furthermore, the relationships between Chinese OID and the two types of perceptions of business practices of compensations and benefits were different: there was little relationship between the PBP and the COIQ sub-scales, whereas PCPs significantly predicted both COIQ dimensions. A possible explanation is the current Chinese workers’ trend of pursuing better pay: According to Leininger (2004), Chinese employees overwhelmingly cited seeking for a better pay as the number one reason why they wanted to leave their company, followed by better career opportunities and better training and development opportunities. Therefore, we concluded that the two dimensions of Chinese OID as measured with the COIQ had distinct predictive validity.

Although not hypothesized, one additional interesting finding is the lack of direct relationships between perceptions of compensation and benefit practices and employees’ UPB, meaning that employees’ appreciation of their company’s (better) compensation and benefits did not necessarily mean that they would choose to act (unethically so) on behalf of their company, which was inconsistent with a common perception in China that money can motivate people to do anything, such as in the Chinese proverb, “If you have money, you could make the devil push the millstone for you.” One possible explanation for this phenomenon is that the antecedents of UPB might be less likely based on employer-employee transactional relationships, such as compensation and benefits, but more likely based on individual and/or organizational characteristics, such as one’s stronger personality trait of Machiavellianism (Castille et al., 2018), one’s own moral identity centrality and one’s perception of their company as having a greater moral identity centrality (Matherne et al., 2018).

#### Chinese Workers’ OID Profiles

Lastly, our LPA showed that workers could be classified into three OID profiles depending on how strongly they identified with their firm. The three groups are (a) those who strongly identified with their organization, showing a higher level of congruence between their feeling and action (i.e., the Strong Identifiers), (b) those who were more reserved in terms of declaring their OID and/or those preferred to stay in the middle, reflecting a popular mindset of Chinese doctrine of the mean (i.e., the Moderate Identifiers), and (c) those who showed a lower emotional identification level with their company, but paradoxically strived to support their company at the same time, probably because those activities might benefit their career no matter how they felt about the organization (i.e., the Action-Oriented Identifiers). Not surprisingly, our further explorations showed differential relationships between the OID profiles and some other relevant organizational constructs (i.e., PCV, perceived company’s compensation and benefit practices, and UPB). Noticeably, the Strong Identifiers not only reported less PCV perception, and perceived their organization’s compensation practices more favorably, but also expressed slightly more willingness to engage in unethical behavior for the good of the company.

Note that the third OID profile of employees (the Action-Oriented Identifiers) might be parallel to an empirical observation in the recent review of the OID literature by Tsuchiya (2017): Some people who do not positively evaluate organization identity still say that their organizational identity exists. The author thus called for further research on OID as a dynamic process. Our preliminary evidence of a divergence between feelings and actions, as well as across the levels of OID among Chinese employees might offer a further breakdown of the concept toward that research focus.

#### Research and Practical Implications

The COIQ has research implications for the field of human resources management in China. Boisot and Child (1999) noticed that skilled Chinese workers, particularly mid-level and senior managers, were not highly loyal to their employers, a consequence of the shortage of working capital in a fast-growing economy like China. For example, 38% of Chinese HR professionals reported a sharp increase of turnovers in their organization (Howard et al., 2007); the average tenure of executive managers was only 1–2 years, and mid- and higher-level managers/leaders frequently left their previous jobs for better career opportunities elsewhere. The turnover rate among Chinese managers was 25% higher than the global average (Gross and Heinold, 2005); 30–40% of senior managers at multinational companies switched jobs every year. At the same time, according to Grant (2006), there were only 1.2 million out of 15.7 million Chinese university graduates being suitable for employment in large multinational companies.

In Chinese culture, in any work relationship between an employee and his or her employer, there is always a component of “instrumentality” or “Li” (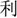; Huang, 1988) and an element of “human sentiment” or “Renqing” (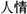; King, 1991). As aforementioned, the former element appears to eclipse the latter in the current Chinese employer-employee relationship because both parties might not expect a long-lasting or stable relationship with each other. Nowadays, most Chinese professionals are in a race for the highest pay and the best job possible (Keeley, 2003). However, IBM China once surveyed their employees about what they considered the most effective motivation factors, and found that most employees still considered non-monetary factors (e.g., work achievement, responsibility, and managerial support) as more important than monetary rewards (Fan, 2006). Facing such a low supply and a high demand in the skilled labor market, human resource managers in Chinese companies or foreign organizations operating in China have experienced the need to identify who are high organizational identifiers among their managers and employees in order to develop appropriate talent-management and retention strategies, which would benefit their companies in the long run. Therefore, the 8-item COIQ provides an appropriate, valid and reliable tool for researchers and practitioners in need of a way to measure individual differences in Chinese OID, which is the first step to extend the literature and examine possible HR interventions.

The findings regarding profiles of Chinese workers in terms of their OID tendency on both dimensions of emotion and action need further verification in future research. Nevertheless, the three profiles of organizational identifiers that we were able to map in Study 2 have evidenced the utility of the COIQ measure, such that employers might be able to use this valid measurement tool to identify and strategically retain high identifiers in their firms. However, as most of the ratings are high on the COIQ behavioral dimension, it might be that our sample did not consist of any workers who might have a high turnover tendency (i.e., reducing their emotional and behavioral commitment), a finding that deserves further investigation.

## General Discussion

The general contributions of our multiple-study field research program were two-fold. First, we tested a bi-dimensional framework of Chinese OID (i.e., affective and behavioral processes), which is both similar to and different from existing Western frameworks (i.e., having a behavioral element but lacking a prominent cognitive element, which might be intertwined with the emotional element instead). We drew an explanation for our findings from the literature of cross-cultural emotional management, such that Chinese workers might display both thought and feeling about their company in the same stroke.

Secondly, we developed, validated and cross-validated the factor structure of the COIQ, a measure of Chinese OID concept, triangulating our previous qualitative findings. Taken together, we concluded that the Chinese OID had its unique characteristics when compared with the Western OID concept, and the COIQ as a measurement instrument demonstrated both reliability and construct validity across our two employee samples.

### Strengths and Limitations

There were several methodological strengths and limitations in our studies. In terms of strengths, we successfully developed and cross-validated the measure of Chinese OID as an emic-etic construct in our series of quantitative studies, establishing the construct validity of Chinese OID (see Messick, 1989). More importantly, we applied the latent profile analysis method to the COIQ data to generate Chinese OID profiles. There is a level of complexity in our findings: Not only were we able to classify our sample into two groups of identifiers based on their declared identification levels (highly or moderately) on both Chinese OID dimensions, but we also found a small group of employees who did not behave uniformly on the two dimensions of the COIQ (i.e., low on affect but high on action). The contradiction in the third OID profile was interestingly consistent with some anecdotal evidence of OID as a dynamic, changing process, on which Tsuchiya (2017) called for more research. To that goal, our bi-dimensional COIQ might provide a measurement vehicle for that research direction in China.

A limitation of the present study was the use of convenience sampling techniques in recruiting participants, possibly resulting in non-representativeness of the findings. However, there was substantial variance in all samples’ relevant characteristics (e.g., job positions, industries, demographics) in our studies.

Another possible limitation is our use of self-report, cross-sectional data which might result in biased findings due to common method variance. However, a cross-sectional design is appropriate for our research goals because we were only interested in individual differences among respondents’ perception of OID at the time when they were employed in their current position in their organization; we were not interested in how their perception of OID would develop or change over time (i.e., time not being one of the study variables), thus a longitudinal research design was not necessary. The cross-sectional design is also practical and ethical for dealing with our research participants: asking respondents about their feelings about and actions toward their organizations might make some respondents reluctant or uncomfortable. In addition, we promised complete anonymity in exchange for participants’ honest responses, which would not have been possible with a longitudinal research design. Nevertheless, we monitored possible common method bias effects and found no significant evidence of such effects. Also, note that the use of self-report survey is appropriate in our study program because we investigated subjects’ opinions and perceptions (Whitley, 2002) and self-report data can be used to empirically test the generalizability of a research model (Fowler, 1998).

### Future Directions

Previous research has indicated that the nature of employment will influence how willingly employees were to identify with their organization (e.g., Zajonc, 1968; Veenstra et al., 2004). Future researchers should focus on possible situational moderators of OID in the workplace, such as the relationship between employees and supervisors, and the nature of tasks and compensations.

On the one hand, most participants in our research program were born after 1980 (often referred to as “post-80” or balinghou in Chinese; Marquardt, 2008). Since they are often the only child in the current China family structure, they are nurtured as “little emperors/empresses” (Emery and Tian, 2010). Consequently, they are more likely to prioritize their personal goals, feelings, and interests over organizational and/or society’s goals and interests (Zhang, 2007).

On the other hand, participants might have been instilled the concept of “guanxi” from early childhood, aware of the critical role of harmony, solidarity, loyalty to supervisors, and obedience in the workplace (Redding, 1982; Lee, 1996). Their personal work experience would also strengthen their awareness that *guanxi* is still important in China. As a result, individuals might display a paradoxical mode of understanding and acting in terms of OID: In our qualitative study prior to the present studies, we additionally found that some workers might emphasize the importance of a family-like atmosphere in the workplace, but at the same time they did not willingly sacrifice their personal interests nor offer more substantial support to their organization. In addition, as mentioned above, the economic transition in China and the adoption of an open-door policy has created a cultural context in which both Western and Eastern value systemsrelevant to organizations co-exist (Matthew, 2000; Guan and Dodder, 2001). Chinese employees may be influenced by such a mixed value system, which in turn increases the complexity of their thinking and behavior. Therefore, future research needs to consider those contextual characteristics.

Given that the two COIQ subscales have distinct predictive validity, and given the profile analytic results in the present study, future research may also focus on testing the characteristics of workers who fits a certain profile (e.g., a Strong Identifier vs. an Action-Oriented Identifier), and how those levels of OID could be related to retention/turnover and other organizational outcomes in China. For instance, the most loyal employee should score high on both dimensions, whereas those score low on both dimensions are most likely to distance themselves from the company. Unclear to researchers is the possible complex pattern of feeling and action of those with a mixture of low and high scores on the two dimensions of emotional and behavior factors.

On the measurement side, the COIQ provided a valid and suitable tool for further research in dis-identification, ambivalent identification, and neutral identification in China, given that Chinese employees might constantly compare and contrast themselves with other workers or between their organization and other firms. Kreiner and Ashforth (2004) posited that OID and disidentification are two distinct constructs. In reality, employees may identify with some aspects of their organization and disidentify with other aspects at the same time (Pratt, 2000; Kreiner and Ashforth, 2004), implying a co-existence of OID, disidentification, ambivalent identification, and neutral identification. Therefore, more research on the mixed nature of OID subconstructs, especially on the negative side of organizational dis-identification, may shed further light on the OID phenomenon in China.

Last but not least is the future research question of whether Chinese OID is a multi-level, emergent organizational phenomenon. Although OID is mainly defined as a cognitive process in the Western literature (e.g., Rousseau, 1998; van Dick, 2001), most research has focused on OID as a static individual characteristic, the level of OID being on a continuum (e.g., higher vs. lower). Yet, OID as a dynamic process might encompass both individual workers’ underlying psychological mechanisms and employees’ interaction with their supervisors’ management style and/or their organizations’ practices of “treating employees well” (Fan, 2006). As such, OID could multiply and become a collective energy throughout a work unit or a company due to strong identifiers’ inspiring their colleagues. In other words, Chinese OID could be studied as a multilevel, emergent organizational behavior. Kozlowski and Klein (2000) write, “A phenomenon is emergent when it originates in the cognition, affect, behaviors, or other characteristics of individuals, is amplified by their interactions and manifests as a higher-level, collective phenomenon” (p. 55). In other words, Chinese OID meets the research criteria proposed by Kozlowski et al. (2013): It originates at an individual’s emotions and actions, and could be developed or ruined over time through a process of observing and interacting with other individuals (peers, supervisors) as well as with institutional factors (e.g., work opportunities; management respecting “face”) in the context of a fierce battle for talents in Corporate China. Future mixed-method, multilevel studies should be conducted to shed further light on the emergence of Chinese workers’ OID in that context.

## Ethics Statement

This study was carried out in accordance with the recommendations of the Jiangxi University of Finance and Economics academic committee with written informed consent from all subjects. All subjects gave written informed consent in accordance with the Declaration of Helsinki. The protocol was approved by the University’s academic committee.

## Author Contributions

JY and H-HN contributed equally to the conceptualization of this research program and the manuscript preparation. XX and XW contributed to the data collection and/or data analyses.

## Conflict of Interest Statement

The authors declare that the research was conducted in the absence of any commercial or financial relationships that could be construed as a potential conflict of interest.
